# State of the Art of microRNAs Signatures as Biomarkers and Therapeutic Targets in Parkinson’s and Alzheimer’s Diseases: A Systematic Review and Meta-Analysis

**DOI:** 10.3390/biomedicines11041113

**Published:** 2023-04-07

**Authors:** Idiberto José Zotarelli-Filho, Bassam Felipe Mogharbel, Ana Carolina Irioda, Priscila Elias Ferreira Stricker, Nathalia Barth de Oliveira, Claudia Sayuri Saçaki, Maiara Carolina Perussolo, Nádia Nascimento da Rosa, Larissa Lührs, Dilcele Silva Moreira Dziedzic, Rogério Saad Vaz, Katherine Athayde Teixeira de Carvalho

**Affiliations:** 1 Advanced Therapy and Cellular Biotechnology in Regenerative Medicine Department, The Pelé Pequeno Príncipe Research Institute & Pequeno Príncipe Faculties, Curitiba 80240-020, Brazil; dr.idibertozotarelli@faceres.com.br (I.J.Z.-F.); bassamfm@gmail.com (B.F.M.); anairioda@gmail.com (A.C.I.); priscilaeferreira@gmail.com (P.E.F.S.); nadianr@gmail.com (N.N.d.R.); laariluhrs@gmail.com (L.L.); dilceledz@gmail.com (D.S.M.D.); 2 Faculty of Medicine of São José do Rio Preto, FACERES., São José do Rio Preto, São Paulo 15090-305, Brazil; 3 UNIFATEB Centro Universitário de Telêmaco Borba, Telêmaco Borba 84266-010, Brazil; saadvaz@gmail.com

**Keywords:** Parkinson’s disease, Alzheimer’s disease, biomarkers, therapeutic target, diagnosis, exosomes, microRNA

## Abstract

Identifying target microRNAs (miRNAs) might serve as a basis for developing advanced therapies for Parkinson’s disease (PD) and Alzheimer’s disease. This review aims to identify the main therapeutic targets of miRNAs that can potentially act in Parkinson’s and Alzheimer’s diseases. The publication research was conducted from May 2021 to March 2022, selected from Scopus, PubMed, Embase, OVID, Science Direct, LILACS, and EBSCO. A total of 25 studies were selected from 1549 studies evaluated. The total number of miRNAs as therapeutic targets evidenced was 90 for AD and 54 for PD. An average detection accuracy of above 84% for the miRNAs was observed in the selected studies of AD and PD. The major signatures were miR-26b-5p, miR-615-3p, miR-4722-5p, miR23a-3p, and miR-27b-3p for AD and miR-374a-5p for PD. Six miRNAs of intersection were found between AD and PD. This article identified the main microRNAs as selective biomarkers for diagnosing PD and AD and therapeutic targets through a systematic review and meta-analysis. This article can act as a microRNA guideline for laboratory research and pharmaceutical industries for treating Alzheimer’s and Parkinson’s diseases and offers the opportunity to evaluate therapeutic interventions earlier in the disease process.

## 1. Introduction

In the scenario of neurodegenerative diseases, Alzheimer’s disease (AD) occupies the first position of the most incident neurodegenerative disease, and in second place is Parkinson’s disease (PD), impacting 1% of the population over 60 years of age [1]. Patients with PD usually have non-motor symptoms, including autonomic nervous system disorders such as constipation, bladder dysfunction, orthostatic hypotension, impaired sleep, and smell, in addition to motor symptoms such as resting tremor, postural instability, gait disturbances, rigidity, and bradykinesia [1].

Otherwise, at the molecular level, the abnormal accumulation of the α-synuclein protein (α-Syn) is related to the degeneration of dopaminergic neurons, with consequent dopamine deficiency [2]. In this sense, the accumulation of α-Syn, the formation of Lewy bodies and Lewy neurites, and their mutations and multiplication are linked to hereditary PD, according to Braak’s hypothesis [3].

As a form of treatment, nucleic acid therapy for PD includes dopamine biosynthetic enzymes for increasing dopamine production or modulate basal ganglia circuitry for alleviating motor symptoms [4], as well as the use of factors trophies in an attempt to increase the survival of dopaminergic neurons [5,6]. Thus, the spotlight is focused on the negative regulation of α-Syn gene expression, highlighting the microRNAs (miRNA) [2]. In this way, at least five genes have been associated with this multigenic disease, including α-synuclein, leucine-rich repeat kinase 2 (LRRK2), parkin, phosphatase and tensin homolog-induced kinase 1 (PINK1), as well as DJ-1 [7], being promising therapeutic targets for the treatment of PD [8].

AD is a progressive neurodegenerative disease characterized by memory loss, multiple cognitive impairments, and changes in personality and behavior. According to the 2018 World Alzheimer’s Report, more patients were diagnosis, and more than 50 million people are estimated to have dementia in 2018. This number is estimated to increase to 82 million by 2030 and 152 million by 2050. However, two-thirds of women and one-third of men are at risk of being diagnosed with AD at some point in their lives [9]. The major risk for developing AD is aging.

In this context, early-onset familial AD affects people younger than 65 years of age, with genetic mutations in the amyloid precursor protein (APP), presenilin 1 (PS1), and presenilin 2 (PS2) genes as the leading cause of familial AD early-onset [9]. Furthermore, the ApoE 4/4 genotype significantly contributes to late-onset disease, and genetic polymorphisms in the CD2AP, EPHA1, and MS4A4/MS4A6E receptors are reported [9]. In addition, predictors such as type 2 diabetes, traumatic brain injury, stroke, and Down Syndrome stand out. Lifestyle, diet, environment, and age can contribute to late-onset AD [10].

In preclinical studies, anatomopathological investigations of AD from mouse brain tissues revealed that multiple findings are related to the pathogenesis of AD, such as defective miRNA regulation, mitochondrial damage, synaptic dysfunction, and amyloid formation and accumulation, as well as the formation of neuritic and neurofibrillary plaques in the brain [10,11,12,13,14,15,16,17,18,19]. Based on these findings, developing innovative therapeutic strategies is imperative, highlighting cell therapy and its products, such as exosomes and miRNAs [20].

In this approach, exosomes are extracellular vesicles that contain proteins, mRNAs, miRNAs, and DNAs [21] and play an essential role in cellular communication through biomolecules. Evidence suggests that MSC-derived exosomes (MSC-EXO) exhibit functions similar to MSCs with low immunogenicity and do not stimulate malignant transformation [22,23,24,25,26,27,28], and they express several cytokines such as Tumor Necrosis Factor-α (TNF-α), Granulocyte-Macrophage Colony Stimulating Factor (GMCSF), and Interleukin (IL)-2, IL-6, IL-8, IL-10, IL-15, IL-1β [29,30,31].

The exosomes and miRNA are involved in cellular communication and are documented as critical therapeutic targets in neurodegenerative diseases [31]. Furthermore, miRNAs function as biomarkers for the early diagnosis of these diseases [32] and can regulate post-transcriptional gene expression by binding to the 3′ untranslated region (UTR) of their target mRNAs [33]. The regulation was mediated by mRNA cleavage/degradation or translation inhibition [34]. In this sense, the altered expression of specific miRNAs in patients with AD and PD points to the important role of miRNAs in the pathogenesis of these diseases and the therapeutic potential [35,36].

The present study aimed to identify, through a systematic review and meta-analysis, the microRNAs related to Parkinson’s and Alzheimer’s diseases, establishing a guideline for developing new therapies.

## 2. Materials and Methods

### 2.1. Study Design

The present study followed the international model of systematic reviews and meta-analyses, following the rules of PRISMA (preferred reporting items for systematic reviews and meta-analyses) (Supplementary Table S1 and Figure S1) [37] and registered in the Institutional Review Board of PROSPERO International of systematic reviews (protocol code CRD354228, 17 August 2022).

Table 1 shows the main variables that were addressed in the present study, according to the designation of the PICOS literary search strategy (Participants; Intervention; Control; Outcomes, and Study Design).

### 2.2. Instruments and Professionals Used for Study Eligibility

The studies were rigorously chosen following the search strategy in Table 1, presented scientific quality according to the GRADE classification [38], and did not present a risk of significant bias, that is, they did not compromise the safety of the data results, according to the Cochrane instrument [39].

For the selection and enrollment of the studies, two independent reviewers performed the research and study selection. Data extraction was performed by reviewer one and was thoroughly reviewed by reviewer two. A third investigator decided on some conflicting points for the final selection of the articles. Only studies reported in English were evaluated.

### 2.3. Eligibility Criteria, Study Quality, and Risk of Bias

According to the recommendations of GRADE [38], the quality of scientific evidence in the studies addressed was classified as high, moderate, low, or very low, according to the risk of evidence bias, sample size, clarity of comparisons, precision, and consistency in the effects of the analyses. High-quality evidence was assigned through seven criteria: (1) In vitro controlled randomized clinical trials (human biological samples); (2) Sample size greater than 15 biological samples; (3) Studies that showed an accuracy (%) of quantitative polymerase chain reaction (qPCR) measurements above 50%; (4) Studies that showed Alzheimer’s and Parkinson’s diseases with a genetic cause and not by transitory or epigenetic effects; (5) Studies that were controlled by biological samples from patients with mild cognitive impairment (MCI), frontotemporal lobar degeneration, DLB (dementia with Lewy bodies), multiple system atrophy, and Progressive Supranuclear palsy; (6) Studies with statistically well-designed results; (7) Studies that were published in indexed journals and had a significant impact factor.

The Cochrane Instrument [39] was adopted to assess the risk of bias in the selected studies, using the Cohen Test to calculate the effect size (Effect Size) versus the inverse of the Standard Error (precision or sample size) to determine the Risk of Bias of the studies using the Funnel Plot.

### 2.4. Data Sources, Research Strategy, and Study Publication Date

The search strategies for the present study were based on the keywords of the medical subject headings (Mesh Terms): Parkinson’s disease; Alzheimer’s disease; Biomarkers; Therapeutic target; Diagnosis; Exosomes; MicroRNA. Search filters designated as clinical studies and clinical studies with biological samples were used. The publication search was developed based on Scopus, PubMed, Embase, OVID, Science Direct, LILACS, and EBSCO. In addition, a combination of the keywords with the Booleans “OR” and “AND” and the “NOT” operator were used to target scientific articles of interest. The title and abstracts were examined under all conditions. Table 2 presents an example of the search structure in PubMed. The same search strategy was used in the other databases.

### 2.5. Statistical Analysis—Meta-Analysis

The statistical programs Minitab 18^®^ (version 18, Minitab, LLC, State College, PA, USA) and OriginPro^®^ 9 (DPR Group, Inc., Northampton, MA, USA) were used. Descriptive statistical analysis was performed for numerical variables, with the mean values, standard deviation, confidence interval (CI), and percentage. The Anderson–Darling (AD) normality test was performed for non-binary numerical variables, adopting *p* > 0.10 as normal (standard). The Cohen test was performed to calculate the effect size (Effect Size). The inverse of the standard error (precision or sample size) was established to determine the risk of bias in the studies using the Funnel Plot. The Heterogeneity Test (Chi-Square Test ≥ X^2^) of the results between the studies was also determined, with *p* < 0.05 and with no statistically significant difference, in the 95% CI, adopting low association codes ≤ 25%, medium association = 25% < X < 50%, and high association ≥ 50%. The One-Way test (ANOVA) was performed between the values of the means of identification accuracy of the microRNAs, adopting the α level lower than 0.05, with a statistically significant difference for the 95% CI. To know the chances of a particular microRNA being identified more than once, the Nominal Logistic Regression analysis test was carried out, adopting a referential group with the Odds Ratio (OR) calculation to know the probability ratio between the analyzed groups, with 95% CI.

## 3. Results

A total of 25 studies (11 studies of Parkinson’s disease (PD) only, 12 studies of Alzheimer’s disease (AD) only, and two studies that presented both AD and PD in the same work) were selected from a total of 1549 evaluated studies (581 (PD) and 968 (AD)), comprising a total of 2160 human participants, a moderate to a high quality of scientific evidence, and an average degree of confidence and a recommendation of 80%, according to the GRADE classification (Supplementary Figure S1). In addition, it was observed that the analyzed studies showed homogeneity in the results in terms of accuracy in identifying samples of AD and PD miRNAs, showing 98.95% (X^2^).

Table 3 shows the results of the Detection Rate (Accuracy (%) or accuracy of miRNA identification by qPCR in each selected study). Through the correlation between the test and control groups in each study, the Chi-Square method (X^2^) test showed that all correlations presented a statistically significant difference, with *p* > 0.05 in the 95% CI, for both AD and PD studies. Table 3 also presents the results of the effect size (Cohen’s Test) and the 1/standard deviation (sample size) to determine the risk of bias in the studies addressed in this work.

**Figure 1:**
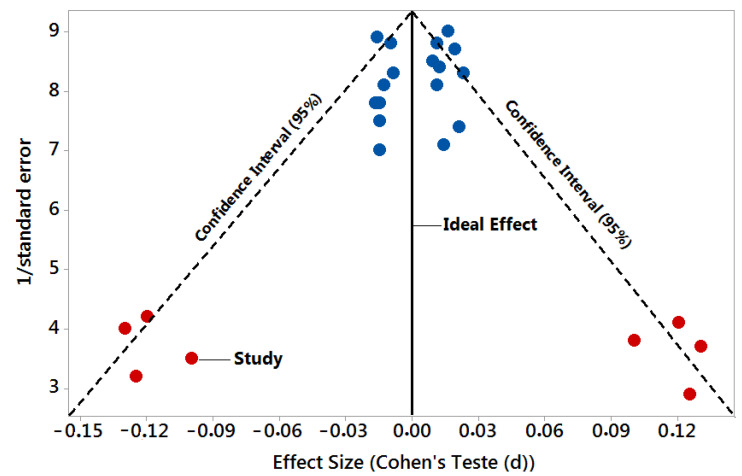
The symmetrical Funnel Plot does not suggest a risk of bias between the small-sample-size studies that are shown at the bottom of the graph. High-confidence and high-recommendation studies are shown above the graph (blue balls).

Figure 1 presents the results of the risk of bias of the studies through the Funnel Plot, showing the calculation of the Effect Size (magnitude of the difference) using the Cohen Test (d). This graph presented a symmetrical behavior, not suggesting a significant risk of bias, both among studies with a small sample size (lower precision, with a total of eight (8) studies), which are shown at the base of the graph (red balls), and studies with a high sample size, with a total of 17 studies, which are presented in the upper region of Figure 1.

Table 4 summarizes the main general findings of each study addressed in this work. Of the 25 studies selected to compose the meta-analysis, only two studies (Burgos et al., 2014 [40] and Nie et al., 2020 [41]) presented results of the quantification of miRNAs for both AD and PD. As evidenced by therapeutic targets, the total of up-regulated and down-regulated miRNAs was 90 for AD and 54 for PD, obtained mainly from CSF, serum, and plasma. Most studies had two types of controls: a control composed of healthy participants, and one composed of participants with mild cognitive impairment (MCI), frontotemporal lobar degeneration, DLB (dementia with Lewy bodies), multiple system atrophy, and paralysis progressive supranuclear.

**Figure 2:**
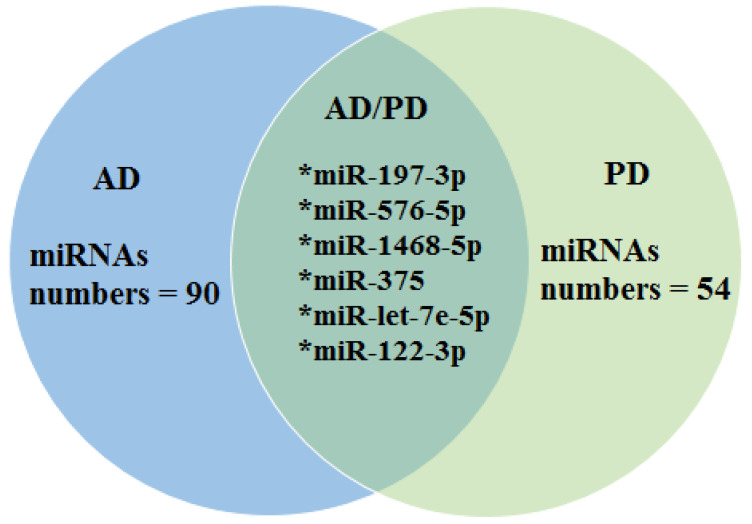
Number and types of miRNAs found at the intersection between Alzheimer’s disease (AD) and Parkinson’s disease (PD). * Common microRNAs in AD and PD (intersection).

Figure 2 shows the number of deregulated miRNAs that were identified in both AD and PD studies. Six miRNAs of intersection were found between AD and PD (miR-197-3p, Mir-576-5p, miR-1468-5p, miR-375, miR-let-7e-5p and miR-122-3p).

Through the Forest Plot graph presented in Table 5 and Table 6 relating to AD, the values distribution of each study’s means and the standard deviation of accuracy (%) concerned the total mean of 84.37 ± 7.94%, in the confidence interval of 95%. Through this, eight studies were identified with accuracy values (%) equal to or above the total average. These eight studies are identified by reference numbers [2,3,4,5,8,11,12,14].

These eight studies were selected to determine their respective types of miRNAs. They presented the highest accuracies (%) in identifying and quantifying the miRNAs, with greater scientific credibility as biomarkers and therapeutic targets in identifying AD, either in the up- or downregulation.

In addition, Tukey’s statistical analysis (One-Way ANOVA) showed that there was no statistically significant difference between the studies with the highest accuracy (%), with *p* > 0.05 in the 95% CI. The study groups presented these results with the same letter, as shown in Table 6.

Table 5 represents the statistical analysis results of the accuracy (%) of identification and quantification by qPCR of miRNAs concerning AD. Fourteen studies were listed, showing each study’s mean and standard deviation of accuracy (%), with a total mean of 84.37 ± 7.94%.

Through the Forest Plot graph presented in Table 7 relating to PD, each study’s mean values and standard deviation of accuracy (%) concerning the total mean value of 84.32 ± 7.15% (CI 95%) were distributed. Thus, seven studies were identified with accuracy values (%) above the total average. These seven results are demonstrated by the studies with reference numbers [2,16,18,21,22,23,25].

These seven studies were selected to determine their respective types of miRNAs. They present the highest accuracy (%) in identifying and quantifying the most scientifically credible miRNAs as biomarkers and therapeutic targets in identifying PD in up- and downregulation. 

Table 7 represents the statistical analysis results of the accuracy (%) of identification and quantification by qPCR of miRNAs concerning PD. A total of thirteen studies were listed, showing each study’s mean and standard deviation of accuracy (%), with a total mean of 84.32 ± 7.15%.

**Figure 3:**
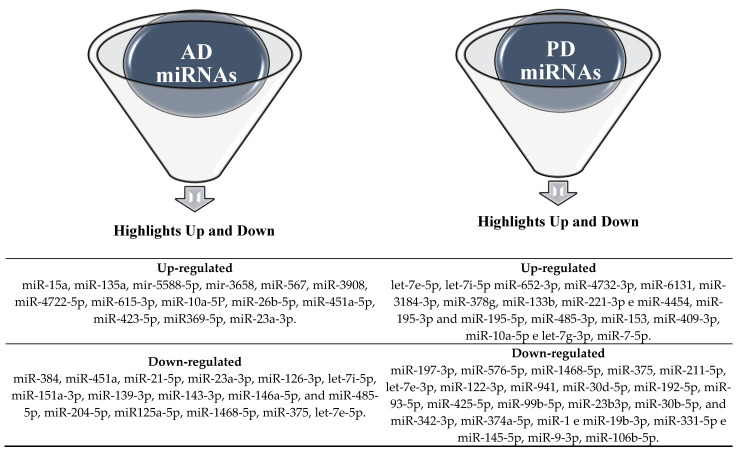
miRNAs above the mean concerning sample identification accuracy. Alzheimer’s disease (AD); Parkinson’s disease (PD).

After identifying the most scientifically reliable miRNAs of the selected studies through the accuracy (%) or precision analysis, as shown in Table 5 and Table 7, the main miRNAs for AD and PD were listed in up- and downregulation, respectively, as shown in Figure 3. 

Additionally, there was no significant difference between studies with higher accuracy (%), according to Tukey’s analysis, with *p* > 0.05 in the 95% CI. The study groups presented these results with the same letter, as shown in Table 8. 

Of the total number of miRNAs identified in the present study, 90 AD and 54 PD, it was statistically analyzed by nominal logistic regression to determine which of these miRNAs had the highest odds (Odds Ratio) of being identified by qPCR. The results showed five (5) miRNAs—miR-26b-5p (up-regulated), miR-615-3p (up-regulated), miR-4722-5p (up-regulated), miR23a-3p (up-regulated), and miR-27b-3p—for AD, with OR = 2.55 (1023-3432) and *p* = 0.004 < 0.05. Regarding PD, the results showed miR-374a-5p (down-regulated), with OR = 2.16 (0.087-3.567) and *p* = 0.0035 < 0.05, as shown in Table 9.

**Figure 4:**
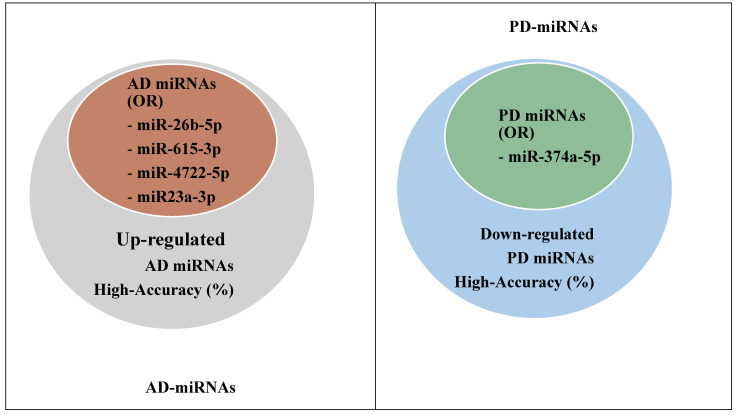
Illustrative scheme showing the inclusion of miRNAs from Table 7 in the respective groups of Figure 3. Alzheimer’s disease (AD); Parkinson’s disease (PD).

Based on the results presented in Table 9, a search was conducted to determine which of these miRNAs are present in the groups of the main AD and PD miRNAs selected by the accuracy criterion (%) shown in Figure 3. The results showed that all miRNAs (AD and PD) that had the highest chances of being identified by qPCR (Table 9) were included in the groups of the main miRNAs of high accuracy (%), except for miR-27b-3p, belonging to the AD group in Table 9, as shown in Figure 4.

## 4. Discussion

Based on the objective of the present study, it was evidenced that the majority of the twenty-five studies of AD and PD presented a mean accuracy in identifying miRNAs by qPCR above 84%, with moderate to strong scientific evidence. These showed greater scientific credibility in the findings of each study, contributing in a tangible way to identifying the main miRNAs as selective biomarkers for the diagnosis of these diseases, as well as therapeutic targets in gene, cellular, and pharmacological treatment.

The present study’s results do not show a risk of bias, both in studies with large and small sample sizes. In addition, two studies (Burgos et al., 2014 [40] and Nie et al., 2020 [41]) presented the results of the quantification of miRNAs for both AD and PD. The total up-regulated and down-regulated miRNAs as biomarkers and therapeutic targets were obtained mainly from CSF, serum, and plasma. Most miRNAs were obtained from serum and plasma, facilitating laboratories worldwide’ work for rapid sampling identification and quantification sampling.

The published studies selected in the present analysis presented mainly two types of controls, one composed of healthy participants and the other composed of participants with mild cognitive impairment (MCI), frontotemporal lobar degeneration, DLB (dementia with Lewy bodies), multiple system atrophy, and Progressive Supranuclear palsy. In addition, studies that presented Alzheimer’s and Parkinson’s diseases with genetic causes and not by transient or epigenetic effects were selected to eliminate the main confounders in accurately identifying miRNAs for AD and PD.

Furthermore, in the studies with better accuracy rates in the identification by qPCR of AD and PD miRNAs, the distribution of the values of the means and standard deviation of the accuracy (%) of each study concerning the values of the total mean of AD and PD was, respectively, 84.37 ± 7.94% and 84.32 ± 7.15%. Among these, eight studies were identified with accuracy values (%) equal to or above the total average for AD, and seven studies were identified for PD in the identification and quantification of miRNAs (up- and downregulated) of greater scientific credibility as biomarkers and therapeutic targets in the identification of these diseases.

Additionally, from the total number of miRNAs identified (90 AD and 54 PD) which have the highest chances (Odds Ratio) of being identified by qPCR, a regression analysis was performed, which indicated five (5) miRNAs—miR-26b-5p (up-regulated), miR-615-3p (up-regulated), miR-4722-5p (up-regulated), miR23a-3p (up-regulated), and miR-27b-3p for AD, with OR = 2.55 (1023–3432) and *p* = 0.004 < 0.05, and only one (1) miRNA related to PD, miR-374a-5p (down-regulated), with OR = 2.16 (0.087–3.567) and *p* = 0.0035 < 0.05. After crossing the information, the results showed that all miRNAs (AD and PD) that presented the highest chances of being identified by qPCR (Table 9) are included in the groups of the prominent miRNAs with high accuracy (%), except for miR-27b-3p, belonging to the AD group of Table 9, as shown in Figure 4 of this study. These findings strongly highlighted the main miRNAs as biomarkers and therapeutic targets for AD and PD, thus contributing to future studies of advanced therapy with anti-miRNAs or antigenic modulation through vectors such as mesenchymal stem cell exosomes [30,31,32], as well as for pharmacological therapies [2,27,28,29].

In this context, exosomes present a potential mechanism for the modulation of pathological α-Syn in the brain, as they can transport proteins and genetic material between cells, including mRNA and miRNA, contributing to the relief of AD and PD symptoms. Furthermore, because of their small size, exosomes can be used as vectors for the delivery of therapeutics [45,46,47,48].

Considering the critical role of α-Syn in PD, it is clear to understand the mechanisms that regulate its expression for therapeutic purposes since the reduced expression of these specific miRNAs can result in high levels of α-Syn in patients with PD. As a corollary of this, miR-7 and miR-153 have been shown to accelerate the degradation of performed α-Syn fibrils [65,66,67,68,69].

Additionally, MiR-205 levels are reduced in the frontal and striate cortex of PD patients, and LRRK2 expression is increased [70]. Genome-wide association studies have also identified variations in miR-4519 and miR-548at-5p related to PD [71]. However, the present study did not recruit this miRNA because it did not present significant accuracy in serum or plasma, given that the purpose of this study was to elect the main miRNAs of rapid and high identification and quantification for the diagnosis and monitoring of diseases and indications of biological and pharmacological direct relevance. 

Based on these findings, it is essential to better understand the common genetic variants associated with AD and PD since most of the genetic risk remains uncharacterized. It is imperative to understand the role of regulatory elements such as miRNAs. The miRNAs relevant to neurodegenerative diseases are related to axonal guidance, apoptosis, and inflammation, so AD and PD likely arise from defects in underlying biological pathways. Furthermore, pathways regulated by APP, L1CAM, and genes from the caspase family may represent promising therapeutic targets of miRNAs in AD and PD, being therapeutic targets of deregulated miRNAs in both disorders [72].

As a corollary, targeting miRNAs offers a potential therapeutic opportunity for AD and PD, highlighting two strategies. The aim of this approach is based on the hypothesis that the downregulation of the specific protein level is a protective therapeutic strategy [73]. In this sense, the miRNA mimics that are used to inhibit the expression of target proteins stand out.

Moreover, miRNA-based therapy involves using anti-miRNA molecules to cause the loss of specific miRNA function [73]. As an example, miRNA-7 targets the 3′-UTR of α-Syn mRNA and facilitates the clearance of α-synuclein aggregates [74], and it exhibits protective effects against MPP+/1-methyl-4-induced toxicity -phenyl-1,2,3,6-tetrahydropyridine (MPTP) [75,76,77,78,79]. Therefore, miRNAs circulating in the blood and other biofluids can be characterized and used as non-invasive diagnostic biomarkers that facilitate early disease detection and the continuous monitoring of AD and PD disease progression. Such screening is essential for understanding which types of miRNAs change in the progression of these diseases and when these changes happen [80]. 

In this way, the results of the present study can act as a guideline for miRNAs for research laboratories and pharmaceutical industries of interest in the possible treatments of AD and PD diseases. Soon, these results may support the diagnosis of these diseases and offer therapeutic interventions earlier in the disease process.

## 5. Conclusions

Based on the findings of this study, it was evident that most Alzheimer’s and Parkinson’s diseases studies showed accuracy in the qPCR identification of miRNAs above the total average, demonstrating greater scientific credibility and solidly contributing to the identification of the main microRNAs as selective biomarkers for the diagnosis of these diseases, as well as therapeutic targets. 

The major signatures were miR-26b-5p, miR-615-3p, miR-4722-5p, miR23a-3p, and miR-27b-3p for AD and miR-374a-5p for PD. There were six miRNAs of intersection between AD and PD: miR-197-3p, Mir-576-5p, miR-1468-5p, miR-375, miR-let-7e-5p, and miR-122-3p. 

This article can act as a microRNA guideline for research laboratories and pharmaceutical industries for treating Alzheimer’s and Parkinson’s diseases and offer the opportunity to evaluate therapeutic interventions earlier in the disease process.

## Figures and Tables

**Table 1:** Publication Search Strategy—PICOS.

PARTICIPANTS	INTERVENTION	CONTROL	OUTCOMES	STUDY DESIGN
Human serum, plasma, and cerebrospinal fluid samples	Peripheral blood and cerebrospinal fluid collection	Biological samples from healthy patients	Main microRNAs as biomarkers and therapeutic targets	In vitro clinical studies

**Table 2:** For an example of the search structure in PubMed, the same search strategy was used in the other databases.

**PUBMED**	AND	**PUBMED**	AND	**PUBMED**	NOT	**PUBMED**
Parkinson’s disease OR Alzheimer’s disease		Alzheimer’s disease and microRNA and miRNA and human and serum and plasma and cerebrospinal fluid		Parkinson’s disease and microRNA and miRNA and human and serum and plasma and cerebrospinal fluid		Review study OR Editorials OR Short communications

Note: The research was conducted on publications from May 2021 to March 2022.

**Table 3:** Results of the Detection Rate (Accuracy (%) of the identification of miRNAs by qPCR in each selected study.

Authors and Date/Variables	Detection Rate (Accuracy (%))	*p*-Value	Effect Size	1/Standard Error
N = 25 Studies	*Test Group* vs. *Control*	*Reference < 0.05*	*Cohen’s Test (d)*	*Precision or Sample Size*
1. Burgos et al. 2014 [39]	73% AD 55% PD	>0.05	0.012	8.4
2. Nie et al. 2020 [40]	84% AD 95% PD	>0.05	−0.010	8.8
3. Bekris et al. 2013 [41]	92% AD	>0.05	−0.013	8.1
4. Liu et al. 2021 [42]	95% AD	>0.05	−0.017	7.8
5. De Felice et al. 2020 [43]	85.7% AD	>0.05	0.021	7.4
6. Zhao et al. 2020 [44]	76% AD	>0.05	−0.016	8.9
7. Denk et al. 2018 [45]	72% AD	>0.05	0.023	8.3
8. Liu et al. 2014 [46]	96% AD	>0.05	−0.100	3.5
9. Galimberti et al. 2014 [47]	82% AD	>0.05	0.100	3.8
10. Soleimani, Pashazadeh, and MotieGhader 2020 [48]	80% AD	>0.05	−0.015	7.5
11. Liu, Xu, and Yu 2022 [49]	87% AD	>0.05	−0.015	7.8
12. Gámez-Valero et al. 2019 [50]	90% AD	>0.05	0.120	4.1
13. Guévremont et al. 2022 [51]	80% AD	>0.05	−0.120	4.2
14. Jia et al. 2021 [52]	90% AD	>0.05	0.130	3.7
15. Grossi et al. 2021 [53]	73.8% PD	>0.05	−0.130	4.0
16. Chen et al. 2021 [54]	91.1% PD	>0.05	0.125	2.9
17. Manna et al. 2021 [55]	75% PD	>0.05	−0.125	3.2
18. Cai et al. 2021 [56]	97% PD	>0.05	0.009	8.5
19. He et al. 2021 [57]	79% PD	>0.05	0.011	8.8
20. Baghi et al. 2021 [58]	79.3% PD	>0.05	0.011	8.1
21. Jiang et al. 2021 [59]	88.6% PD	>0.05	−0.009	8.3
22. Lin et al. 2021 [60]	88.1% PD	>0.05	0.014	7.1
23. Gui et al. 2015 [61]	85.6% PD	>0.05	−0.015	7.0
24. Vallelunga et al. 2019 [62]	82% PD	>0.05	0.019	8.7
25. Starhof et al. 2019 [63]	88% PD	>0.05	0.016	9.0

Note: Cohen’s Test (d) = 0.020 indicates a small effect, d = 0.050 indicates a medium effect, and d = 0.080 indicates a large effect.

**Table 4:** Results of the Detection Rate (Accuracy (%) of the identification of miRNAs by qPCR in each selected study. Alzheimer’s disease (AD); Parkinson’s disease (PD).

Authors/Study Data	Sample Size (n) (Human Participants)	Disease Type Alzheimer’ Disease (AD) and/or Parkinson’ Disease (PD)	Sample Type	Numbers and Types of miRNAs
1. Burgos et al. 2014 [40]	69 AD 67 PD 78 healthy controls	AD/PD	CSF and Serum (*postmortem*)	AD-Serum: Up-regulated: miR-34b-3p, miR-219-2-3p, miR-34c-5p, miR-34b-5p, miR-135a-5p Down-regulated: miR-182-5p, miR-21-5p, miR-375 AD-CSF: Down-regulated: N = 41 miRNAs (demonstrated in the supplementary material) PD-CSF: Up-regulated: miR-19a-3p, miR-19b-3p, let-7g-3p Down-regulated: miR-132-5p, miR-485-5p, miR-127-3p, miR-128, miR-409-3p, miR-433, miR-370, miR-431-3p, miR-873-3p, miR-136-3p, miR-212-3p, miR-10a-5p, miR-1224-5p, miR-4448 PD (Serum): Up-regulated: miR-338-3p, 30e-3p, 30a-3p Down-regulated: miR-16-2-3p, 1294
2. Nie et al. 2020 [41]	34 healthy controls, 5 AD donors, and 7 PD donors	AD and PD	Plasma	AD: Up-regulated: miR-423-5p, miR369-5p, miR-23a-3p Down-regulated: miR-204-5p, miR125a-5p, miR-1468-5p, miR-375, let-7e-5p PD: Up-regulated: let-7e-5p, let-7i-5p miR-652-3p, miR-4732-3p, miR-6131, miR-3184-3p, miR-378g Down-regulated: miR-197-3p, miR-576-5p, miR-1468-5p, miR-375, miR-211-5p, let-7e-3p, miR-122-3p, miR-941, miR-30d-5p, miR-192-5p, miR-93-5p, miR-425-5p, miR-99b-5p
3. Bekris et al. 2013 [42]	21 AD 21 healthy controls	AD	CSF, Plasma (during life); Cerebellum and Hippocampus were obtainedat autopsy.	Up-regulated: miR-15a (Plasma high levels)
4. Liu et al. 2021 [43]	198 AD 30 healthy controls	AD	LCR Serum	Up-regulated: miR-135a
5. De Felice et al. 2020 [44]	18 AD 18 mild cognitive impairment	AD	LCR	Up-regulated: hsa-mir-5588-5p, hsa-mir-3658, hsa-mir-567 e hsa-mir-3908 Highlight: hsa-mir-567 (Blood, LCR, and Serum)
6. Zhao et al. 2020 [45]	32 AD 51 healthy controls 13 mild cognitive impairment	AD	Serum	Up-regulated: mir-346, mir-345-5p, mir-122-3p, mir-1291, mir-640, mir-650, mir-1285-3p, mir-1299, mir-1267 Down-regulated: mir-208b-3p, mir-499a-5p, mir-206
7. Denk et al. 2018 [46]	48 AD 44 healthy controls 48 frontotemporal lobar degeneration	AD	LCR Serum	Up-regulated: miR-320a and miR-26b-5p
8. Liu et al. 2014 [47]	45 AD 22 MCI 50 healthy controls	AD	LCR Serum	Down-regulated: miR-384
9. GalimberTi et al. 2014 [48]	10 AD 8 healthy controls	AD	LCR Serum	Down-regulated: miR-125b, miR-23a, miR-26b
10. Soleiman, Pashazadeh, and MotieGhader 2020 [49]	145 AD 80 mild cognitive impairment (MCI) 104 healthy controls	AD	LCR Serum	Up-regulated: miR-615-3p, miR-4722-5p, miR-4768-3p, miR-1827, miR-940 e miR-30b-3p
11. Liu, Xu and Yu 2022 [50]	33 AD 33 healthy controls	AD	Serum	Up-regulated: miR-4722-5p e miR-615-3p
12. Gámez-Valero et al. 2019 [51]	10 AD 18 DLB (dementia with Lewy bodies) 15 healthy controls	AD	Plasma	Down-regulated: hsa-miR-451a e hsa-miR-21-5p, hsa-miR-23a-3p, hsa- miR-126-3p, hsa-let-7i-5p e hsa-miR-151a-3p
13. Guévremont et al. 2022 [52]	65 AD 74 MCI 89 healthy controls	AD	Plasma	Down-regulated: miR-27a-3p, miR-27b-3p e miR-324-5p Up-regulated: miR-122-5p, miR-132-3p, miR-193b-3p, miR-320a-3p, miR-365-3p, miR-885-5p
14. Jia et al. 2021 [53]	Pilot study (21 controls; 23 AD3), followed by the second (216 controls; 190 AD) and third groups (153 controls; 151 AD). (139 controls; 155 AD; Amnestic mild cognitive impairment, 55 (aMCI); 51 VaD; 53 PDD; 53 bvFTD; 52 DLB)	AD	Serum	Down-regulated: miR-139-3p, miR-143-3p, miR-146a-5p, miR-485-5p Up-regulated: miR-10a-5P, miR-26b-5p e miR-451a-5p
15. Grossi et al. 2021 [54]	15 PD 14 healthy controls	PD	Plasma	Up-regulated: miR-34a-5p
16. Chen et al. 2021 [55]	151 PD 21 Patients with multiple system atrophy 138 healthy controls	PD	Plasma	Up-regulated: miR-133b, miR-221-3p e miR-4454
17. Manna et al. 2021 [56]	40 PD 20 Progressive Supranuclear Palsy 33 healthy controls	PD	Serum	Up-regulated: miR-21-3p, miR-22-3p e miR-223-5p
18. Cai et al. 2021 [57]	5 PD 7 healthy controls	PD	Plasma	Down-regulated: miR-23b3p, miR-30b-5p, miR-342-3p Up-regulated: miR-195-3p and miR-195-5p
19. He et al. 2021 [58]	72 PD 31 healthy controls	PD	Serum	Up-regulated: hsa-miR-374a-5p, hsa-miR-374b-5p, hsa-miR-199a-3p, hsa-miR-28-5p, hsa-miR-22-5p e hsa-miR-151a-5p
20. Baghi et al. 2021 [59]	20 PD 20 healthy controls	PD	Serum	Up-regulated: miR-193b
21. Jiang et al. 2021 [60]	68 PD 50 healthy controls	PD	Serum	Down-regulated: miR-374a-5p
22. Lin et al. 2021 [61]	92 PD 64 healthy controls	PD	Serum	Up-regulated: miR-485-3p
23. Gui et al. 2015 [62]	47 PD 27 healthy controls	PD	LCR	Down-regulated: miR-1 e miR-19b-3p Up-regulated: miR-153, miR-409-3p, miR-10a-5p e let-7g-3p
24. Vallelunga et al. 2019 [63]	56 PD 49 Multiple System Atrophy 50 healthy controls	PD	Plasma; Serum; LCR	Up-regulated: miR-30c-5p and miR148b-5p
25. Starhof et al. 2019 [64]	37 PD; 29 atypical Parkinson’sdisorder; 32 atypical Parkinson’s (AP) spectrum; 23 healthy controls.	PD	LCR	Up-regulated: miR-7-5p Down-regulated: miR-331-5p e miR-145-5p, miR-9-3p, miR-106b-5p

**Table 5:** Results of the statistical analysis of the accuracy (%) of identification and quantification by qPCR of miRNAs concerning Alzheimer’s disease (AD), with *p* > 0.05 and no statistically significant difference, at 95% CI.

Studies (AD)	Accuracy (%) Mean	StDev	Mean = 84.37 ± 7.94%	95% CI
1 [39]	71.667	1.528	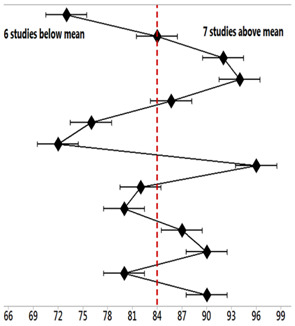	(70.276; 73.058)
2 [40]	83.667	1.528		(82.276; 85.058)
3 [41]	91.500	0.500		(90.109; 92.891)
4 [42]	94.500	0.500		(93.109; 95.891)
5 [43]	85.467	1.365		(84.076; 86.858)
6 [44]	75.500	0.500		(74.109; 76.891)
7 [45]	72.167	0.764		(70.776; 73.558)
8 [46]	96.333	1.528		(94.942; 97.724)
9 [47]	81.167	0.764		(79.776; 82.558)
10 [48]	80.167	0.764		(78.776; 81.558)
11 [49]	87.333	1.528		(85.942; 88.724)
12 [50]	91.000	1.000		(89.609; 92.391)
13 [51]	80.333	1.528		(78.942; 81.724)
14 [52]	90.333	1.528		(88.942; 91.724)

**Table 6:** Tukey’s statistical analysis among studies with the highest accuracy (%), with *p* > 0.05 at 95% CI. The study groups presented these results with the same letter.

Studies	Grouping								
**8**	**A**								
**4**	**A**	**B**							
**3**		**B**	**C**						
**12**		**B**	**C**						
**14**			**C**	**D**					
**11**				**D**	**E**				
**5**					**E**	**F**			
**2**						**F**	**G**		
**9**							**G**		
**13**							**G**		
**10**							**G**		
**6**								**H**	
**7**								**H**	**I**
**1**									**I**

Note: Means that do not share a letter are significantly different, with *p* < 0.05 (CI95%).

**Table 7:** Results of the statistical analysis of the accuracy (%) of identification and quantification by qPCR of miRNAs concerning PD, with *p* > 0.05 and no statistically significant difference, at CI 95%.

Studies (PD)	Mean	StDev	Mean = 84.32 ± 7.15%	95% CI
15 [53]	73.867	1.102	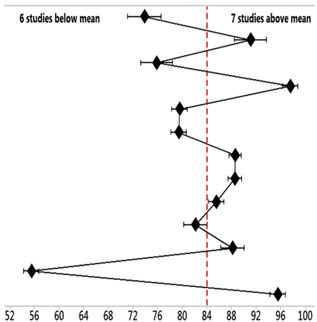	(73.030; 74.703)
16 [54]	91.067	1.050		(90.230; 91.903)
17 [55]	75.833	1.041		(74.997; 76.670)
18 [56]	97.533	0.503		(96.697; 98.370)
19 [57]	79.567	0.513		(78.730; 80.403)
20 [58]	79.433	0.513		(78.597; 80.270)
21 [59]	88.600	0.400		(87.763; 89.437)
22 [60]	88.533	0.451		(87.697; 89.370)
23 [61]	85.533	0.503		(84.697; 86.370)
24 [62]	82.167	0.764		(81.330; 83.003)
25 [63]	88.167	0.764		(87.330; 89.003)
1 [39]	55.500	0.500		(54.663; 56.337)
2 [40]	95.500	0.500		(94.663; 96.337)

**Table 8:** Tukey’s statistical analysis among studies with the highest accuracy (%), with *p* > 0.05 at 95% CI. The study groups presented these results with the same letter.

Studies	Grouping								
**18**	**A**								
**2**	**A**								
**16**		**B**							
**21**			**C**						
**22**			**C**						
**25**			**C**						
**23**				**D**					
**24**					**E**				
**19**						**F**			
**20**						**F**			
**17**							**G**		
**15**							**G**		
**1**								**H**	

Note: Means that do not share a letter are significantly different, with *p* < 0.05 (CI95%).

**Table 9:** Results of nominal logistic regression analysis to identify which miRNAs have the highest odds (Odds Ratio) of being identified by qPCR, with *p* < 0.05 and statistical significance at CI 95%. Alzheimer’ Disease (AD); Parkinson’ Disease (PD).

AD/PD	miRNAs	Odds Ratio (OR)/ *p*-Value (95% CI)
AD	miR-26b-5p miR-615-3p miR-4722-5p miR23a-3p miR-27b-3p	OR = 2.55 (1.023–3.432); *p* = 0.004 < 0.05
PD	miR-374a-5p	OR = 2.16 (0.087–3.567); *p* = 0.0035 < 0.05

## Data Availability

All data generated or analyzed during this study are included in this submitted article.

## Supplementary Materials

The following supporting information can be downloaded at: https://www.mdpi.com/article/10.3390/biomedicines11041113/s1, Figure S1: Flowchart showing the article selection process. PRISMA 2020; Table S1: Prisma 2020 Checklist [81].

## References

[1] Titova N., Qamar M.A., Chaudhuri K.R. (2017). The Nonmotor Features of Parkinson’s Disease. Int. Rev. Neurobiol..

[2] Nakamori M., Junn E., Mochizuki H., Mouradian M.M. (2019). Nucleic Acid-Based Therapeutics for Parkinson’s Disease. Neurotherapeutics.

[3] Braak H., Del Tredici K., Rub U., de Vos R.A., Jansen Steur E.N., Braak E. (2003). Staging of brain pathology related to sporadic Parkinson’s disease. Neurobiol. Aging.

[4] Hamza T.H., Zabetian C.P., Tenesa A., Laederach A., Montimurro J., Yearout D., Kay D.M., Doheny K.F., Paschall J., Pugh E. (2010). Common genetic variation in the HLA region is associated with late-onset sporadic Parkinson’s disease. Nat. Genet..

[5] Dawson T.M., Dawson V.L. (2003). Molecular pathways of neurodegeneration in Parkinson’s disease. Science.

[6] Gasser T. (2009). Molecular pathogenesis of Parkinson disease: Insights from genetic studies. Expert Rev. Mol. Med..

[7] Simon-Sanchez J., Schulte C., Bras J.M., Sharma M., Gibbs J.R., Berg D., Paisan-Ruiz C., Lichtner P., Scholz S.W., Hernandez D.G. (2009). Genome-wide association study reveals genetic risk underlying Parkinson’s disease. Nat. Genet..

[8] Ma L., Wei L., Wu F., Hu Z., Liu Z., Yuan W. (2013). Advances with microRNAs in Parkinson’s disease research. Drug Des. Dev. Ther..

[9] Selkoe D.J. (2001). Alzheimer’s disease results from the cerebral accumulation and cytotoxicity of amyloid beta-protein. J. Alzheimer’s Dis..

[10] Mattson M.P. (2004). Pathways towards and away from Alzheimer’s disease. Nature.

[11] LaFerla F.M., Green K.M., Oddo S. (2007). Intracellular amyloid-beta in Alzheimer’s disease. Nat. Rev. Neurosci..

[12] Swerdlow R.H., Burns J.M., Khan S.M. (2010). The Alzheimer’s disease mitochondrial cascade hypothesis. J. Alzheimer’s Dis..

[13] Reddy P.H., Manczak M., Mao P., Calkins M.J., Reddy A.P., Shirendeb U. (2010). Amyloid-beta and mitochondria in aging and Alzheimer’s disease: Implications for synaptic damage and cognitive decline. J. Alzheimer’s Dis..

[14] Mao P., Manczak M., Calkins M.J., Truong Q., Reddy T.P., Reddy A.P., Shirendeb U., Lo H.H., Rabinovitch P.S., Reddy P.H. (2012). Mitochondria-targeted catalase reduces abnormal APP processing, amyloid β production, and BACE1 in a mouse model of Alzheimer’s disease: Implications for neuroprotection and lifespan extension. Hum. Mol. Genet..

[15] Kumar S., Reddy P.H. (2016). Are circulating microRNAs peripheral biomarkers for Alzheimer’s disease?. Biochim. Biophys. Acta.

[16] Reddy P.H. (2017). A critical assessment of research on neurotransmitters in Alzheimer’s disease. J. Alzheimer’s Dis..

[17] Kumar S., Reddy P.H. (2018). MicroRNA-455-3p as a potential biomarker for Alzheimer’s disease: An update. Front. Aging Neurosci..

[18] Abeliovich A., Flint M. (2006). Beal, Parkinsonism genes: Culprits and clues. J. Neurochem..

[19] Yaghoubi Y., Movassaghpour A., Zamani M., Talebi M., Mehdizadeh A., Yousefi M. (2019). Human umbilical cord mesenchymal stem cells derived-exosomes in diseases treatment. Life Sci..

[20] Baharlooi H., Nouraei Z., Azimi M., Moghadasi A.N., Tavassolifar M.J., Moradi B., Sahraian M.A., Izad M. (2020). Umbilical cord mesenchymal stem cells as well as their released exosomes suppress proliferation of activated PBMCs in multiple sclerosis. Scand. J. Immunol..

[21] Théry C., Boussac M., Véron P., Ricciardi-Castagnoli P., Raposo G., Garin J., Amigorena S. (2001). Proteomic analysis of dendritic cell-derived exosomes: A secreted subcellular compartment distinct from apoptotic vesicles. J. Immunol..

[22] Mears R., Craven R.A., Hanrahan S., Totty N., Upton C., Young S.L., Patel P., Selby P.J., Banks R.E. (2004). Proteomic analysis of melanoma-derived exosomes by two-dimensional polyacrylamide gel electrophoresis and mass spectrometry. Proteomics.

[23] Gastpar R., Gehrmann M., Bausero M.A., Asea A., Gross C., Schroeder J.A., Multhoff G. (2005). Heat shock protein 70 surface-positive tumor exosomes stimulate migratory and cytolytic activity of natural killer cells. Cancer Res..

[24] Futter C.E., White I.J. (2007). Annexins and endocytosis. Traffic.

[25] Staubach S., Razawi H., Hanisch F.G. (2009). Proteomics of MUC1-containing lipid rafts from plasma membranes and exosomes of human breast carcinoma cells MCF-7. Proteomics.

[26] Yim N., Ryu S.W., Choi K., Lee K.R., Lee S., Choi H., Kim J., Shaker M.R., Sun W., Park J.-H. (2016). Exosome engineering for efficient intracellular delivery of soluble proteins using optically reversible protein-protein interaction module. Nat. Commun..

[27] Xu W., Yang Z., Lu N. (2016). From pathogenesis to clinical application: Insights into exosomes as transfer vectors in cancer. J. Exp. Clin. Cancer Res..

[28] Valadi H.H., Ekstrom K., Bossios A., Sjostrand M., Lee J.J., Lotvall J.O. (2007). Exosome mediated transfer of mRNAs and microRNAs is a novel mechanism of genetic exchange between cells. Nat. Cell Biol..

[29] Zhu Z., Zhang Y., Zhang Y., Zhang H., Liu W., Zhang N., Zhang X., Zhou G., Wu L., Hua K. (2019). Exosomes derived from human umbilical cord mesenchymal stem cells accelerate growth of VK2 vaginal epithelial cells through MicroRNAs in vitro. Hum. Reprod..

[30] Zhang B., Shen L., Shi H., Pan Z., Wu L., Yan Y., Zhang X., Mao F., Qian H., Xu W. (2016). Exosomes from Human Umbilical Cord Mesenchymal Stem Cells: Identification, Purification, and Biological Characteristics. Stem Cells Int..

[31] Pinnell J.R., Cui M., Tieu K. (2020). Exosomes in Parkinson disease. J. Neurochem..

[32] Vassileff N., Cheng L., Hill A.F. (2020). Extracellular vesicles—Propagators of neuropathology and sources of potential biomarkers and therapeutics for neurodegenerative diseases. J. Cell Sci..

[33] He L., Hannon G.J. (2004). MicroRNAs: Small RNAs with a big role in gene regulation. Nat. Rev. Genet..

[34] Surguchov A., Peplow P.V., Martinez B., Gennarelli T.A. (2022). Biomarkers in Parkinson’s Disease. Neurodegenerative Diseases Biomarkers.

[35] Junn E., Mouradian M.M. (2010). MicroRNAs in neurodegenerative disorders. Cell Cycle.

[36] Junn E., Mouradian M.M. (2012). MicroRNAs in neurodegenerative diseases and their therapeutic potential. Pharmacol. Ther..

[37] Page M.J., Moher D., Bossuyt P.M., Boutron I., Hoffmann T.C., Mulrow C.D., Shamseer L., Tetzlaff J.M., Akl E.A., Brennan S.E. (2021). PRISMA 2020 explanation and elaboration: Updated guidance and exemplars for reporting systematic reviews. BMJ.

[38] Balshem H., Helfand M., Schünemann H.J., Oxman A.D., Kunz R., Brozek J., Vist G.E., Falck-Ytter Y., Meerpohl J., Norris S. (2011). Grade guidelines: 3 ratng the quality of evidence. J. Clin. Epidemiol. Md. Height.

[39] Higgins J.P.T., Thomas J., Chandler J., Cumpston M., Li T., Page M.J., Welch V.A., Cochrane (2022). Cochrane Handbook for Systematic Reviews of Interventions Version 6.3 (Updated February 2022). www.training.cochrane.org/handbook.

[40] Burgos K., Malenica I., Metpally R., Courtright A., Rakela B., Beach T., Shill H., Adler C., Sabbagh M., Villa S. (2014). Profiles of extracellular miRNA in cerebrospinal fluid and serum from patients with Alzheimer’s and Parkinson’s diseases correlate with disease status and features of pathology. PLoS ONE.

[41] Nie C., Sun Y., Zhen H., Guo M., Ye J., Liu Z., Yang Y., Zhang X. (2020). Differential Expression of Plasma Exo-miRNA in Neurodegenerative Diseases by Next-Generation Sequencing. Front. Neurosci..

[42] Bekris L.M., Lutz F., Montine T.J., Yu C.E., Tsuang D., Peskind E.R., Leverenz J.B. (2013). MicroRNA in Alzheimer’s disease: An exploratory study in brain, cerebrospinal fluid and plasma. Biomarkers.

[43] Liu C.G., Meng S., Li Y., Lu Y., Zhao Y., Wang P.C. (2021). MicroRNA-135a in ABCA1-labeled Exosome is a Serum Biomarker Candidate for Alzheimer’s Disease. Biomed. Environ. Sci..

[44] De Felice B., Montanino C., Oliva M., Bonavita S., Di Onofrio V., Coppola C. (2020). MicroRNA Expression Signature in Mild Cognitive Impairment Due to Alzheimer’s Disease. Mol. Neurobiol..

[45] Zhao X., Kang J., Svetnik V., Warden D., Wilcock G., David Smith A., Savage M.J., Laterza O.F. (2020). A Machine Learning Approach to Identify a Circulating MicroRNA Signature for Alzheimer Disease. J. Appl. Lab. Med..

[46] Denk J., Oberhauser F., Kornhuber J., Wiltfang J., Fassbender K., Schroeter M.L., Volk A.E., Diehl-Schmid J., Prudlo J., Danek A. (2018). Specific serum and CSF microRNA profiles distinguish sporadic behavioural variant of frontotemporal dementia compared with Alzheimer patients and cognitively healthy controls. PLoS ONE.

[47] Liu C.G., Wang J.L., Li L., Wang P.C. (2014). MicroRNA-384 regulates both amyloid precursor protein and β-secretase expression and is a potential biomarker for Alzheimer’s disease. Int. J. Mol. Med..

[48] Galimberti D., Villa C., Fenoglio C., Serpente M., Ghezzi L., Cioffi S.M., Arighi A., Fumagalli G., Scarpini E. (2014). Circulating miRNAs as potential biomarkers in Alzheimer’s disease. J. Alzheimer’s Dis..

[49] Soleimani Zakeri N.S., Pashazadeh S., MotieGhader H. (2020). Gene biomarker discovery at different stages of Alzheimer using gene co-expression network approach. Sci. Rep..

[50] Liu Y., Xu Y., Yu M. (2022). MicroRNA-4722-5p and microRNA-615-3p serve as potential biomarkers for Alzheimer’s disease. Exp. Ther. Med..

[51] Gámez-Valero A., Campdelacreu J., Vilas D., Ispierto L., Reñé R., Álvarez R., Armengol M.P., Borràs F.E., Beyer K. (2019). Exploratory study on microRNA profiles from plasma-derived extracellular vesicles in Alzheimer’s disease and dementia with Lewy bodies. Transl. Neurodegener..

[52] Guévremont D., Tsui H., Knight R., Fowler C.J., Masters C.L., Martins R.N., Abraham W.C., Tate W.P., Cutfield N.J., Williams J.M. (2022). Plasma microRNA vary in association with the progression of Alzheimer’s disease. Alzheimer’s Dement. (Amst.).

[53] Jia L., Zhu M., Yang J., Pang Y., Wang Q., Li Y., Li T., Li F., Wang Q., Li Y. (2021). Prediction of P-tau/Aβ42 in the cerebrospinal fluid with blood microRNAs in Alzheimer’s disease. BMC Med..

[54] Grossi I., Radeghieri A., Paolini L., Porrini V., Pilotto A., Padovani A., Marengoni A., Barbon A., Bellucci A., Pizzi M. (2021). MicroRNA 34a 5p expression in the plasma and in its extracellular vesicle fractions in subjects with Parkinson’s disease: An exploratory study. Int. J. Mol. Med..

[55] Chen Q., Deng N., Lu K., Liao Q., Long X., Gou D., Bi F., Zhou J. (2021). Elevated plasma miR-133b and miR-221-3p as biomarkers for early Parkinson’s disease. Sci. Rep..

[56] Manna I., Quattrone A., De Benedittis S., Vescio B., Iaccino E., Quattrone A. (2021). Exosomal miRNA as peripheral biomarkers in Parkinson’s disease and progressive supranuclear palsy: A pilot study. Park. Relat. Disord..

[57] Cai M., Chai S., Xiong T., Wei J., Mao W., Zhu Y., Li X., Wei W., Dai X., Yang B. (2021). Aberrant Expression of Circulating MicroRNA Leads to the Dysregulation of Alpha-Synuclein and Other Pathogenic Genes in Parkinson’s Disease. Front. Cell Dev. Biol..

[58] He S., Huang L., Shao C., Nie T., Xia L., Cui B., Lu F., Zhu L., Chen B., Yang Q. (2021). Several miRNAs derived from serum extracellular vesicles are potential biomarkers for early diagnosis and progression of Parkinson’s disease. Transl. Neurodegener..

[59] Baghi M., Yadegari E., Rostamian Delavar M., Peymani M., Ganjalikhani-Hakemi M., Salari M., Nasr-Esfahani M.H., Megraw T.L., Ghaedi K. (2021). MiR-193b deregulation is associated with Parkinson’s disease. J. Cell Mol. Med..

[60] Jiang X., Xiao L., Jiang X., Li G., Lu Z. (2021). Screening of Parkinson’s Differential MicroRNA Based on GEO Database and Its Clinical Verification. Biomed. Res. Int..

[61] Lin X., Wang R., Li R., Tao T., Zhang D., Qi Y. (2021). Diagnostic Performance of miR-485-3p in Patients with Parkinson’s Disease and its Relationship with Neuroinflammation. Neuromol. Med..

[62] Gui Y., Liu H., Zhang L., Lv W., Hu X. (2015). Altered microRNA profiles in cerebrospinal fluid exosome in Parkinson disease and Alzheimer disease. Oncotarget.

[63] Vallelunga A., Iannitti T., Dati G., Capece S., Maugeri M., Tocci E., Picillo M., Volpe G., Cozzolino A., Squillante M. (2019). Serum miR-30c-5p is a potential biomarker for multiple system atrophy. Mol. Biol. Rep..

[64] Starhof C., Hejl A.M., Heegaard N.H.H., Carlsen A.L., Burton M., Lilje B., Winge K. (2019). The biomarker potential of cell-free microRNA from cerebrospinal fluid in Parkinsonian Syndromes. Mov. Disord..

[65] Kingsbury A.E., Daniel S.E., Sangha H., Eisen S., Lees A.J., Foster O.J. (2004). Alteration in alpha-synuclein mRNA expression in Parkinson’s disease. Mov. Disord..

[66] Dachsel J.C., Lincoln S.J., Gonzalez J., Ross O.A., Dickson D.W., Farrer M.J. (2007). The ups and downs of alpha-synuclein mRNA expression. Mov. Disord..

[67] Grundemann J., Schlaudraff F., Haeckel O., Liss B. (2008). Elevated alpha-synuclein mRNA levels in individual UV-laser-microdissected dopaminergic substantia nigra neurons in idiopathic Parkinson’s disease. Nucleic Acids Res..

[68] Junn E., Lee K.W., Jeong B.S., Chan T.W., Im J.Y., Mouradian M.M. (2009). Repression of alpha-synuclein expression and toxicity by microRNA-7. Proc. Natl. Acad. Sci. USA.

[69] Doxakis E. (2010). Post-transcriptional regulation of alpha-synuclein expression by mir-7 and mir-153. J. Biol. Chem..

[70] Cho H.J., Liu G., Jin S.M., Parisiadou L., Xie C., Yu J., Sun L., Ma B., Ding J., Vancraenenbroeck R. (2013). MicroRNA-205 regulates the expression of Parkinson’s disease-related leucine-rich repeat kinase 2 protein. Hum. Mol. Genet..

[71] Ghanbari M., Darweesh S.K., de Looper H.W., Van Luijn M.M., Hofman A., Ikram M.A., Franco O., Erkeland S.J., Dehghan A. (2016). Genetic Variants in MicroRNAs and Their Binding Sites Are Associated with the Risk of Parkinson Disease. Hum. Mutat..

[72] Sadlon A., Takousis P., Alexopoulos P., Evangelou E., Prokopenko I., Perneczky R. (2019). miRNAs Identify Shared Pathways in Alzheimer’s and Parkinson’s Diseases. Trends Mol. Med..

[73] Wen M.M. (2016). Getting miRNA Therapeutics into the Target Cells for Neurodegenerative Diseases: A Mini-Review. Front. Mol. Neurosci..

[74] Choi D.C., Yoo M., Kabaria S., Junn E. (2018). MicroRNA-7 facilitates the degradation of alpha-synuclein and its aggregates by promoting autophagy. Neurosci. Lett..

[75] Meister G., Landthaler M., Dorsett Y., Tuschl T. (2004). Sequence-specific inhibition of microRNA- and siRNA-induced RNA silencing. RNA.

[76] Choi D.C., Chae Y.J., Kabaria S., Chaudhuri A.D., Jain M.R., Li H., Mouradian M.M., Junn E. (2014). MicroRNA-7 protects against 1-methyl-4-phenylpyridinium-induced cell death by targeting RelA. J. Neurosci..

[77] Chaudhuri A.D., Kabaria S., Choi D.C., Mouradian M.M., Junn E. (2015). MicroRNA-7 Promotes Glycolysis to Protect against 1-Methyl-4-phenylpyridinium-induced Cell Death. J. Biol. Chem..

[78] Kabaria S., Choi D.C., Chaudhuri A.D., Jain M.R., Li H., Junn E. (2015). MicroRNA-7 activates Nrf2 pathway by targeting Keap1 expression. Free Radic Biol. Med..

[79] Chaudhuri A.D., Choi D.C., Kabaria S., Tran A., Junn E. (2016). MicroRNA-7 Regulates the Function of Mitochondrial Permeability Transition Pore by Targeting VDAC1 Expression. J. Biol. Chem..

[80] Mushtaq G., Greig N.H., Anwar F., Zamzami M.A., Choudhry H., Shaik M.M., Tamargo I.A., Kamal M.A. (2016). miRNAs as Circulating Biomarkers for Alzheimer’s Disease and Parkinson’s Disease. Med. Chem..

[81] Page M.J., McKenzie J.E., Bossuyt P.M., Boutron I., Hoffmann T.C., Mulrow C.D., Shamseer L., Tetzlaff J.M., Akl E.A., Brennan S.E. (2021). The PRISMA 2020 statement: An updated guideline for reporting systematic reviews. BMJ.

